# Confirmatory factor analysis of the proxy version of Kidscreen-27 and relationships between health-related quality of life dimensions and body mass index and physical activity in young schoolchildren

**DOI:** 10.1016/j.pmedr.2020.101210

**Published:** 2020-09-16

**Authors:** Kirsti Riiser, Sølvi Helseth, Knut-Andreas Christophersen, Kristin Haraldstad

**Affiliations:** a Department of Physiotherapy, Faculty of Health, OsloMet - Oslo Metropolitan University, PO Box 4 St. Olavsplass, N-0130 Oslo, Norway; b Department of Nursing and Health Promotion, Faculty of Health, OsloMet - Oslo Metropolitan University, PO Box 4 St. Olavsplass, N-0130 Oslo, Norway; c Department of Political Science, University of Oslo, PO Box 1097, Blindern, N-0317 Oslo, Norway; d Department of Health and Nursing Sciences, University of Agder, PO Box 422, N-4604 Kristiansand, Norway

**Keywords:** Young children, Kidscreen, Proxy report, Physical well-being, Physical activity, Body mass index

## Abstract

The aim of the study was to perform a confirmatory factor analysis (CFA) to investigate the psychometric properties of the proxy version of Kidscreen-27 in order to determine whether the instrument can be used to assess health-related quality of life (HRQoL) in young children (five to six years of age). Furthermore, we aimed to examine the relationships between the HRQoL dimensions and the body mass index (BMI), physical activity (PA), age, and gender. Altogether, 276 children from schools in eastern Norway were included (September 2016). HRQoL was measured using the Kidscreen-27 proxy version. CFA was conducted to examine the factorial validity of the five-dimension instrument. Structural equation modelling was used to estimate the relationship between the independent variables and the HRQoL subscales that showed an acceptable fit; physical well-being, social support and peers, and school environment. PA was positively, and BMI negatively associated with physical well-being (p < 0.5). Parents of the youngest children reported more negatively on the school environment subscale (p < 0.5). The full 27-item proxy version of Kidscreen should be used with caution for children as young as five to six years as two of the subscales were found to have unsatisfactory factor loadings. The physical well-being, the social support and peers, and the school environment subscales can provide valid and valuable data for research and practice. Even though the associations are small, it is worrying that adverse relationships between PA and BMI and physical well-being are detectable in such a young sample as included here.

## Highlights

• The full Kidscreen-27 should be used with caution for young children.
• Physical activity was positively associated with physical well-being.
• Body mass was negatively associated with physical well-being.
• Compared to older children, the youngest may have more negative school experiences.

## 1 Introduction

Health-related quality of life (HRQoL) is considered to be an important outcome by researchers as well as practitioners. HRQoL measures aim to assess various aspects of a respondent’s functional status and well-being, including the physical, psychological, and social domains (Ravens-Sieberer, 2014). A growing number of studies have examined the HRQoL in general child and adolescent populations, as well as in specific groups; however, less is known about the HRQoL among young children in early or middle childhood (<8 years). The majority of general child and adolescent populations’ studies show that HRQoL decreases with age, which makes a case for including younger children in studies in order to compare them to children of an older age. It is particularly interesting to gain knowledge about the well-being of children when they enter grade school, as the transition from kindergarten can be an overwhelming experience. Although children in general populations tend to report relatively high quality of life, previous studies of children and adolescents aged 8–18 have shown that there are variations in scores and that some children report low HRQoL (Haraldstad et al., 2017, Michel, 2009). More specifically it has been documented that HRQoL varies with gender, especially among adolescents (Michel, 2009, Helseth et al., 2015, Otto, 2017), but whether gender differences are present in younger populations still needs to be investigated. Previous studies have also shown that in general samples of children and adolescents aged 8–18, body mass is an important contributor for explaining the differences in HRQoL subscales, particularly the subscales of physical well-being, psychological well-being, and self-perception (4, 6). However, knowledge is lacking about whether similar results can be found among younger children. Possessing such knowledge can help teachers and school health representatives to increase their awareness of children’s psychosocial and physical issues, to identify children at risk at an early stage, and to develop targeted interventions to strengthen their HRQoL.

There is a range of validated instruments for assessing HRQoL among children, self-reported as well as proxy-reported. Young children are partly dependent on their parents to judge and report on their well-being. Attempts have been made to investigate the differences between the children’s and the parents’ perspectives concerning children’s HRQoL. Studies show that there are differences and that the directions of these differences vary (Helseth et al., 2015, Berman, 2016). Hence, when interpreting proxy data, it is essential to understand the results as the parents’ knowledge of their children’s well-being. Kidscreen is the most commonly administered HRQoL instrument for studies on children and adolescents in Europe. The instrument exists in three versions (52-, 27-, and 10-item) and is applicable to children from 8 to 18 years of age, either in the form of a self-report or a proxy-report (The KIDSCREEN Group Europe, 2006). Kidscreen-27 is designed to consist of a 5-dimensional structure; *physical well-being, psychological well-being, parent relations and autonomy, social support and peers, and school environment* and was developed as an alternative for clinical use (The KIDSCREEN Group Europe, 2006). Studies that have investigated the cross-cultural comparability and psychometric properties of the Kidscreen-27 self-report instrument were found satisfactory (Ravens-Sieberer, 2007, Andersen, 2016), while others have shown unacceptable fits (Shannon, 2017, Ng, 2015). Neither the self-report version nor the proxy-report version of Kidscreen are commonly included in studies on children younger than eight years of age, with some exceptions which both refer to studies of clinical samples (Razafimahefa-Raoelina, 2016, Stea, 2016). However, we are familiar with the fact that Kidscreen-27 is increasingly being used as a patient-reported outcome to guide practice. In some Norwegian municipalities, it is applied as a supplement to support the public health nurses’ conversations with children and parents during routine checks for school starters. To the best of our knowledge, the psychometric properties of the proxy version of the instrument have not been investigated in a non-clinical population younger than eight years of age.

The aim of the present article is 1) to investigate the psychometric properties of the proxy version of Kidscren-27 in order to determine whether the instrument could be used to assess HRQoL in young children (five to six years old) and 2) to further examine the relationships between the HRQoL dimensions and the body mass index (BMI), physical activity (PA), age, and gender.

## 2 Methods

### 2.1 Study participants

The cross-sectional data included in this article were collected at baseline of a study of young children’s physical activity play in after-school programs (ASP) (ClinicalTrials; NCT02954614) (Riiser, 2017). Schools from municipalities in three counties in eastern Norway were invited to participate. The school administrators gave their consent to participation before the parents of all first graders (aged 5–6) attending the ASPs were informed about the study and asked for a signed consent on behalf of their children. Altogether 14 ASPs agreed to participate. They were situated in urban as well as more rural areas and varied in number of students. Parents representing 456 first graders (5–6 years) gave consent to participation and 447 provided valid e-mail addresses along with the consent. There were no exclusion criteria except not being able to read and write Norwegian. A unique invitation for the electronic questionnaire containing the Kidscreen-27 instrument was sent to each given address of which 63% completed the questionnaire. This resulted in a sample of 276 children who were included in the confirmatory factor analyses while 261 met the requirements for valid days of PA registrations. The sampling procedure and a protocol for the RCT is published elsewhere (Riiser, 2017).

### 2.2 Outcomes

*Children’s age and birth quartile*, indicating whether a child was born in the January–March, April–June, July–September, or October–December period, were obtained from the school’s registers prior to the beginning of the study.

*Body mass index* (BMI) was calculated individually based on the assessment of height and weight (BMI = kg/m2). The height and weight were measured using the weight and stadiometer available at the school nurses’ offices. The children wore light clothing and no shoes. Trained nurses or physiotherapists were responsible for taking the measurements. Age and gender specific BMI cut-off values according to the International Obesity Task Force and the Norwegian clinical guidelines were used to categorize the children as normal weight (BMI < 25), overweight (25 ≤ BMI < 30), and obese (BMI ≥ 30) (Cole, 2000, Directorate, 2010).

*Physical activity* was objectively assessed using the ActiGraph GT3X accelerometer (ActigraphTM LLC, *Pensacola, US) during the ASP time over a period of one week (Monday to Friday) in October 2016. The children wore the monitors for the entirety of their ASP attendance. The mean daily time spent in an ASP was 2.7 h. Valid accelerometer data files consisted of at least two days with at least 60 min accelerometer wear time which is in line with previous studies (Cradock, 2016, Magnusson, 2011). Raw accelerometer files were downloaded and then reintegrated in 10 s epochs to detect the intermittent activity patterns of the children. The results are given as counts per minute (CPM). A more detailed description of the process of obtaining, screening, and analyzing the accelerometer data has been provided in previous papers (Riiser, 2017, Riiser, 2019).

*Health-related quality of life* (HRQoL) was measured using the Kidscreen-27 proxy report version. In the present study, the questionnaire was completed electronically by one of the parents of each participating child. The Kidscreen-27 represents the 10 dimensions from the full 52-item version merged into five dimensions: *physical well-being* (five items), *psychological well-being* (seven items), *autonomy and parent relation* (seven items), *social support and peers* (four items), and *school environment* (four items). The proxy version consists of similar items as the child version but additionally asks the parents how they think their child is feeling. In the present trial, the parents were requested to ensure that the responses reflected their children’s perspectives. Each item was rated on a five-point Likert scale, indicating either the intensity of an attitude or the frequency of a behavior or feeling. Examples of an item from the physical well-being dimension is: “When thinking about the last week, has your child been able to run well?” which is answered on a scale from 1 (not at all) to 5 (extremely). Another item example from the psychological well-being dimension is “When thinking about the last week, has your child been satisfied with herself/himself as she/he is?” which is answered on a scale from 1 (never) to 5 (always). Negatively worded items were rotated according to the manual (The KIDSCREEN Group Europe, 2006). The dimension scores were transformed to a 0–100-point scale, with 100 indicating the best quality of life and 0 indicating the worst.

### 2.3 Statistical analyses

Initially we aimed to evaluate the model fit for the hypothesized five-dimensional structure and the alternative seven-dimensional structure of the proxy Kidscreen-27 version. As previous research has indicated, there is mixed support for the original five-factor model of the self-report Kidscreen-27 version and, hence, a 7-factor model has been suggested (Ng, 2015). The three *dimensions physical well-being*; *social* support and peers and *school environment* are equal to the original 5-dimensions structure. The remaining two (*psychological well-being* and *autonomy and parent relations*), each include items from three different subscales from the 52-item version. Confirmatory factor analyses (CFA) was conducted (AMOS 25) to examine the factor structure of the instrument. We based the assessments of the model fit on the following indices: The χ^2^/df, the *p-*value, the root mean square error of approximation (RMSEA), the Tucker–Lewis index (TLI), the goodness-of-fit index (GFI), and the comparative fit index (CFI). We used the following critical values to conclude: 1) Good fit; χ^2^/df < 2, p *> 0.*05; RMSEA < 0.05; and TLI, GFI, and CFI > 0.95 and 2) Acceptable fit; χ2/df < 3, p > 0.05; 0.05 < RMSEA < . 08; and 0.90 < TLI, GFI, and CFI < 0.95 (Byrne, 2010, Kline, 2005, Arbuckle and Wothke, 1999). Structural equation modelling (AMOS 25) was also used to estimate the relationships between the four independent variables (gender, age, BMI, and PA) and the dependent variables (the HRQoL subscales showing acceptable fit). In the model, birth quartile was treated as a dummy coded categorical variable and the BMI and PA as continuous.

## 3 Results

### 3.1 Results of confirmatory factor analysis (CFA)

The fit-values shown in Table 1, Table 2 were near the boundary of acceptable fit. For the five-factor model, the χ^2^/df < 3 indicates that the fit is acceptable even if p < 0.001. The RMSEA was 0.048, which is close to the border between a good and an acceptable fit. The TLI (0.912), GFI (0.880), and CFI (0.916) were all close to, or above, 0.90 and indicate an acceptable fit for the original five-factor model. The fit values for the seven-factor model were only slightly better and thus we considered it unfounded to replace the original model. The fit indices for the two models are presented in Table 1.

**Table 1:** Summary of the fit indices for the Kidscreen 5- and 7- factor models, N = 276.

Model	χ^2^ / df	*p*	CFI	GFI	TLI	RMSEA
5-factor model	510.29/310 = 1.64	<0.001	0.929	0.880	0.919	0.048
7-factor model	469.01/298 = 1.57	<0.001	0.939	0.891	0.928	0.046

**Table 2:** Summary of the fit indices for the Kidscreen-27 subscales, N = 276.

Kidscreen-27	χ^2^/df	*p*	CFI	GFI	TLI	RMSEA
Physical well-being	16.45/5 = 3.29	<0.01	0.976	0.976	0.952	0.091
Psychological well-being	31.24/11 = 2.84	<0.01	0.951	0.967	0.907	0.082
Autonomy and parent relation	26.46/13 = 2.04	<0.05	0.972	0.971	0.954	0.061
Social support & peers	3.56/2 = 1.78	0.17	0.997	0.994	0.992	0.053
School environment	2.05/1 = 2.05	0.15	0.998	0.996	0.985	0.062

When we assessed the fit of each of the factors in the five-factor model separately, we found that, for two of the factors—psychological well-being and autonomy and parent relation—the fit indices were close to acceptable (Table 2). However, the factor loadings for several of the items were weak. For the psychological well-being subscale, four of seven items had factor loadings below 0.36. For the autonomy and parent relations subscale, three of seven items had loadings below 0.38. The school environment and the autonomy and parent relation subscales had strong residual correlations, while the psychological well-being subscale had a cluster of moderate and even residual correlations, indicating that these items represented separate factors.

The fit indices CFI, GFI, and TLI were acceptable for the physical well-being, social support and peers, and school environment subscales. Factor loadings with approximately the same value indicate homogeneous indicators and substance relevance, which was almost the case here (Table 2).

The factor loadings varied between 0.47 and 0.80 (physical well-being), between 0.62 and 0.86 (social support and peers) and between 0.65 and 0.86 (school environment), as shown in Table 3. The three factors were thus included as dependent variables in a structural equation model, with gender, age, BMI, and PA as independent variables. For the factors psychological well-being (0.17, 0.81) and autonomy and parent relation (0.19, 0.72) the indicators do not meet the criterion for factor loadings. Therefore, these two factors were not included in the structural equation model. The reliability scores for the subscales were good for physical well-being (0.820), social support and peers (0.868) and school environment (0.818), acceptable for psychological well-being (0.707) and close to acceptable for autonomy and parent relation (0.688).

**Table 3:** Item factor loadings for Kidscreen −27.

Kidscreen-27*	Factor loadings
Physical well-being	
*In general, how would your child rate his/her health?*	0.47
*…felt fit an well?*	0.80
*…been physically active?*	0.76
*…been able to run well?*	0.78
*…felt full of energy?*	0.64
Psychological well-being	
*…felt that life was enjoyable?*	0.60
*…been in a good mood?*	0.80
*…had fun?*	0.81
*…felt sad?*	0.32
*…felt so bad that he/she didn’t want to do anything?*	0.24
*…felt lonely?*	0.17
*…been happy with the way he/she is?*	0.36
Autonomy and parent relation	
*…had enough time for him/herself?*	0.38
*…been able to do the things that he/she wants to do in his/her free time?*	0.60
*…felt that his/her parents had enough time for him/her?*	0.64
*…felt that his/her parent(s) treated him/hear fairly?*	0.72
*…been able to talk to his/her parent(s) when he/she wanted to?*	0.61
*… had enough money to do the same things as his/her friends?*	0.19
*…felt that he/she had enough money for his/her expenses?*	0.22
Social support & peers	
*…spent time with his/her friends?*	0.62
*…had fun with his/her friends?*	0.84
*…and his/her friends helped each other?*	0.86
*…been able to rely on his/her friends?*	0.85
School environment	
*…been happy at school?*	0.65
*…got on well at school?*	0.77
*…been able to pay attention?*	0.86
*…got along well with his/her teachers?*	0.73

* The following instructions were given to the parents (according to the Kidscreen manual): “Please answer the following questions to the best of your knowledge, ensuring that the answers you give reflect the perspective of your child. Please try to remember your child’s experiences over the last week.” All questions except the first were opened by “Has your child…” (The KIDSCREEN Group Europe, 2006).

### 3.2 Relationships between the physical well-being, the social support and peers, and the school environment HRQoL-dimensions and PA, gender, age, and BMI

From the sample of 276 children who were included in the CFA, not all provided valid accelerometer and BMI data (missing data 9,5%). Thus, 261 children were included in the model that investigated the associations between the HRQoL dimensions and the gender, age, BMI, and objectively measured PA. The sample was evenly distributed for gender and birth quartiles and 52 children (20%) were categorized as overweight or obese. The mean values (SD) for physical well-being, social support and peers, and school environment were 82.2 (15.6), 71.8 (16.8), and 84.0 (13.3), respectively.

**Fig. 1:**
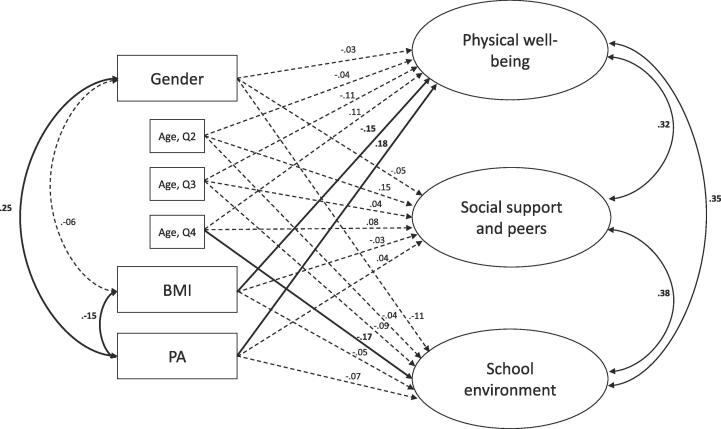
Model displaying the associations (standardized regression coefficients) between gender, age, BMI, and PA. For visual simplicity, measurement terms are not shown. All solid paths are statistically significant, the curves indicate correlations, and the straight lines indicate controlled bivariate relations (possible causal relations). The model was estimated with N = 261 children from Eastern Norway (September 2016).

The model investigating the relationships between the study variables (Fig. 1) showed that the BMI was significantly negatively associated with physical well-being (p < 0.05), while the PA during ASP time was significantly positively associated with physical well-being (p < 0.01). Age (being born in the fourth quartile) was statistically negatively associated with the school dimension (p > 0.05). Moreover, gender and BMI were statistically negatively associated with PA (Fig. 1). Although some of the path coefficients were statistically significant, they contributed relatively modestly to the predicted variance. The R2 for each of the dependent variables was 0.07 (physical well-being), 0.02 (social support and peers), and 0.05 (school environment).

## 4 Discussion

The aim of the present article was to explore some of the psychometric properties of the proxy version of Kidscreen-27 for children five to six years of age. As noted earlier, Kidscreen was originally developed for older children (8–18 years of age). However, the instrument is becoming increasingly popular in research and is also gaining attention in practice. Thus, it was relevant to perform psychometric testing to investigate whether Kidscreen is also suitable for assessing HRQoL in younger populations. We found that the original 5-factor model of the Kidscreen-27 proxy version showed a close to acceptable fit. A modified 7-factor model proved to only slightly be better. When investigating the factors from the original Kidscreen-27 dimensions separately, two of the original five subscales were found to have unsatisfactory factor loadings. These two dimensions, psychological well-being and autonomy and parent relations, each include items from three different subscales from the 52-item version (The KIDSCREEN Group Europe, 2006). Previous research has also found that these factors load into independent factors partly corresponding with the original Kidscreen-52 structure (Ng, 2015), while others do not report similar issues (Robitail, 2007). Based on the CFA results in this study, the full 27-item proxy version of Kidscreen, including the two subscales of psychological well-being and autonomy and parents relations, should be used with caution for children as young as five to six years of age. Further testing is needed to conclude whether these dimensions are valid when assessing HRQoL for such a young sample. It might also be that these scales contain items that are less relevant to young children. However, the remaining three dimensions—physical well-being, social support and peers, and school environment—showed acceptable fit and can provide valid and valuable data for research and practice.

Evidence from studies of parent–child agreement on Kidscreen have shown mixed results (Helseth et al., 2015, Berman, 2016). In general, there is moderate to good correspondence between the child’s self‐report and the parent’s proxy‐report in non-clinical populations. The agreement is found to be better on domains reflecting PA, functioning, and symptoms in comparison to non-observable domains, such as emotional and social HRQoL (Eiser and Morse, 2001). Thus, it has been suggested that self‐ and proxy‐report should be interpreted together in order to complement each other (Meyer, 2016). Cognitive science research shows that even young children can meet the demands of answering questionnaires about their own health and well-being (Riley, 2004). This is however, dependent of the design of the instrument and also the possibility of being assisted in completing the questionnaire. In our opinion, it is not feasible for children as young as six years of age to complete the Kidscreen self-report version on their own, as they would struggle to comprehend the wording of the questions and to understand the rating of answers on a scale even if the items were read aloud. We argue that proxy reports are valuable in their own right because the parents’ perceptions of their children’s feelings contribute to their reactions and actions.

Due to a lack of studies that include samples of children younger than eight years of age, the European Kidscreen-27 proxy report norm for children aged 8–11 provides the most relevant comparable data (The KIDSCREEN Group Europe, 2006). In the present study, the mean score for all three subscales—physical well-being, social support and peers, and school environment—were either above or well above the 50th percentile for European norms and gender was not associated with either of the three subscales. This came as no surprise because it has been thoroughly documented in previous studies that HRQoL starts to decrease at a later time, when children enter adolescence, and that this decrease is most prominent among girls (Michel, 2009, Meade and Dowswell, 2016). In the current study we found a small association between the age and the school environment subscales. Compared to their oldest classmates, children born in the last quartile of a year (October–December) were rated statistically significantly lower in the school dimension. In Norway, the academic year begins in August, enrolling all children born in the same calendar year. At this point in a year, the oldest children are halfway through their seventh year and the youngest halfway through their sixth. It has been shown that school start age has a significant impact on school performance, both short and long term (Solli, 2017). Being older and more mature is assumed to affect learning outcomes as well as the self-esteem and social standing of the child (Solli, 2017, Thompson et al., 2004). Kidscreen asks the parents to rate questions related to both school performance and well-being, such as: “Has your child been happy at school?” and “Has your child got on well at school?” (The KIDSCREEN Group Europe, 2006). We have not been able to find other studies with an HRQoL variation based on age at school start. We argue that the associations found in our study, although weak, are interesting because they relate to similar research and should thus be further investigated.

In the present study, which originated from a larger intervention study on PA among first graders in ASP, it was particularly interesting to investigate the potential relations between the HRQoL dimensions and the objectively measured PA. The relations between the PA and HRQoL are not extensively investigated in children and, particularly, not in the youngest populations. A recent review that included samples <18 years of age found a small to negligible statistically significant association between PA and HRQoL in descriptive studies and a small to medium positive effect of PA on HRQoL in intervention studies (Marker et al., 2018). The great majority of studies in this review included children older than five years of age. Thus, the current study represents a valuable supplement to this field of research. Despite our study measuring the PA during a limited period of the day, we managed to capture a small positive association between the objectively measured PA and the proxy-reported physical well-being dimension of HRQoL. This implies that feeling fit, full of energy, being physically active, and able to run well is related to the PA intensity in situations in which the children are provided with opportunities for self-chosen physical activity play, as is the case in most Norwegian ASPs.

Another noteworthy finding of this study was the negative association between the BMI and the physical well-being HRQoL dimension. This corresponds with previous studies that included older children, which show that overweight and obese children and adolescents have a significantly lower HRQoL than their normal weight peers. The strongest HRQoL impairments are repeatedly found to emerge on the physical well-being scale (Ottova, 2012, Buttitta, 2014). Remembering that this scale includes items that are more easily observed by others, it is likely that parents are better able to register how their children’s weight affects their physical and motor abilities. However, it is important to notice that the physical well-being dimension in Kidscreen asks questions that are primarily related to activities that require cardiorespiratory fitness or to weight-bearing activities—“Has your child been physically active (e.g., running, climbing, biking)?” and “Has your child been able to run well?” Such activities are more strenuous for individuals who carry more of their own weight. If the physical well-being scale had contained questions about activities that are, to a lesser degree, influenced by body weight (e.g., strength, stabilizing skills, and object control), then a negative association between the physical well-being and the BMI may not have been found.

The negative impact of being overweight was also observable for the PA intensity during the ASP stay. The relationship between the PA and BMI is described in a previous publication and is not discussed here (Riiser, 2019). However, it is worth reflecting on the fact that these associations between the PA, BMI, and physical well-being, associations that grow in strength with age, are detectable in such a young sample as the one included in the present study. This underlines that it is critical to devote increased attention to less active young children who are overweight and to support their engagement in physical activities they can master and enjoy.

The current study includes cross-sectional data and its results must not be interpreted as causational—e.g., that PA causes physical well-being. Physical well-being may just as well be a predictor of PA. Moreover, the generalizability of the results is limited because the sample was self-selected and relatively small. Lastly, the children’s PA was measured only during their ASP stay and their PA for the remainder of the day was not taken into consideration. Despite these limitations, this study has valuable strengths because very few other studies have previously investigated HRQoL in young children. The CFA conducted here represents one of the first important attempts to investigate whether proxy versions of Kidscreen are reliable instruments for use in younger populations.

## 5 Conclusions

The present study found that the full 27-item proxy version of Kidscreen should be used with caution for children as young as six years of age. However, its physical well-being, social support and peers, and school environment subscales can provide valid and valuable data for both research and practice. Although the associations are small, it is worrying that the adverse associations between PA and BMI and the physical well-being are detectable in such a young sample as included in the present study. More studies are needed to rigorously assess the psychometric properties of the Kidscreen-27 proxy version for young children.

## Funding

This project is funded by the Norwegian Fund for Postgraduate Training in Physiotherapy.

## CRediT authorship contribution statement

**Kirsti Riiser:** Conceptualization, Funding acquisition, Project administration, Methodology, Formal analysis, Visualization, Writing - original draft. **Sølvi Helseth:** Conceptualization, Writing - review & editing. **Knut-Andreas Christophersen:** Formal analysis, Visualization, Writing - review & editing. **Cand. polit:** . **Kristin Haraldstad:** Conceptualization, Writing - review & editing.

## Declaration of Competing Interest

The authors declare that they have no known competing financial interests or personal relationships that could have appeared to influence the work reported in this paper.
